# Supplementary material for: An unsupervised XAI framework for dementia detection with context enrichment
Source: Sci Rep. 2025 Nov 12;15:39554. doi: 10.1038/s41598-025-26227-2 (PMC12612133; doi:10.1038/s41598-025-26227-2)
Supplement: Supplementary file 1 — Supplementary Material 1 [file 41598_2025_26227_MOESM1_ESM.docx]

Supplementary Material for ‘An Unsupervised Explainable AI Framework for Dementia Detection with Context Enrichment’

**Devesh Singh^1,36^, Yusuf Brima^1^, Fedor Levin^1^, Martin Becker^2^, Bjarne Hiller^2^, Andreas Hermann^1,3^, Irene Villar-Munoz^4,5^, Lukas Beichert^6^, Alexander Bernhardt^7,8^, Katharina Buerger^7,9^, Michaela Butryn^10,11^, Peter Dechent^31^, Emrah Düzel^10,11^, Michael Ewers^7,9^, Klaus Fliessbach^12,13^, Silka D. Freiesleben^4,5,37^, Wenzel Glanz^10,11^, Stefan Hetzer^32^, Daniel Janowitz^9^, Doreen Görß^1,14^, Ingo Kilimann^1,14^, Okka Kimmich^12^, Christoph Laske^15,16^, Johannes Levin^7,8,17^, Andrea Lohse^39^, Falk Luesebrink^10^, Matthias Munk^15,18^, Robert Perneczky^7,17,19,20^, Oliver Peters^4,5^, Lukas Preis^5,37^, Josef Priller^4,5,21,22,38^, Johannes Prudlo^1,23^, Diana Prychynenko^4,5^, Boris S. Rauchmann^19,24,25^, Ayda Rostamzadeh^26^, Nina Roy-Kluth^12^, Klaus Scheffler^34^, Anja Schneider^12,13^, Louise Droste zu Senden^37^, Björn H. Schott^27,28,35^, Annika Spottke^12,33^, Matthis Synofzik^6,15^, Jens Wiltfang^27,28,29^, Frank Jessen^12,26,30^, Marc-André Weber^36^, Stefan J. Teipel^1,14^, and Martin Dyrba*^1^**

^1^ German Center for Neurodegenerative Diseases (DZNE), Rostock/Greifswald, Germany

^2^ Institute for Visual and Analytic Computing, University of Rostock, Germany

^3^ Translational Neurodegeneration Section “Albrecht Kossel”, Department of Neurology, University Hospital Rostock, Rostock, Germany

^4^ German Center for Neurodegenerative Diseases (DZNE), Berlin, Germany

^5^ Charité – Universitätsmedizin Berlin, corporate member of Freie Universität Berlin and Humboldt Universität zu Berlin, Department of Psychiatry and Neuroscience, Hindenburgdamm 30, 12203 Berlin, Germany.

^6^ Division Translational Genomics of Neurodegenerative Diseases, Hertie Institute for Clinical Brain Research and Center of Neurology, University of Tübingen, Tübingen, Germany.

^7^ German Center for Neurodegenerative Diseases (DZNE), Munich, Germany

^8^ Department of Neurology, University Hospital of Munich, Ludwig-Maximilians-Universität (LMU) Munich, Munich, Germany

^9^ Institute for Stroke & Dementia Research, University Hospital, LMU Munich, Germany

^10^ German Center for Neurodegenerative Diseases (DZNE), Magdeburg, Germany

^11^ Institute for Cognitive Neurology and Dementia Research, Faculty of Medicine, University Hospital Magdeburg, Magdeburg, Germany

^12^ German Center for Neurodegenerative Diseases (DZNE), Bonn, Germany

^13^ Department for Neurodegenerative Diseases and Gerontopsychiatry, University of Bonn, Bonn, Germany

^14^ Department of Psychosomatic Medicine, Rostock University Medical Center, Rostock, Germany

^15^ German Center for Neurodegenerative Diseases (DZNE), Tübingen, Germany

^16^ Section for Dementia Research, Hertie Institute for Clinical Brain Research, Department of Psychiatry and Psychotherapy, University Hospital Tübingen, Tübingen, Germany

^17^ Munich Cluster for Systems Neurology (SyNergy), Munich, Germany

^18^ Department of Psychiatry and Psychotherapy, University Hospital Tübingen, Tübingen, Germany

^19^ Department of Psychiatry and Psychotherapy, University Hospital, LMU Munich, Munich, Germany

^20^ Ageing Epidemiology Research Unit, School of Public Health, Faculty of Medicine, Imperial College London, London, United Kingdom

^21^ Department of Psychiatry and Psychotherapy, School of Medicine and Health, Technical University of Munich, Germany

^22^ University of Edinburgh and UK Dementia Research Institute, Edinburgh, United Kingdom

^23^ Department of Neurology, University Medical Centre, Rostock, Germany

^24^ Sheffield Institute for Translational Neuroscience, The University of Sheffield, Sheffield, United Kingdom

^25^ Department of Neuroradiology, University Hospital, LMU Munich, Germany

^26^ Department of Psychiatry, University of Cologne, Medical Faculty, Cologne, Germany

^27^ German Center for Neurodegenerative Diseases (DZNE), Goettingen, Germany

^28^ Department of Psychiatry and Psychotherapy, University Medical Center Goettingen, Goettingen, Germany

^29^ Neurosciences and Signaling Group, Institute of Biomedicine (iBiMED), Department of Medical Sciences, University of Aveiro, Aveiro, Portugal

^30^ Cologne Excellence Cluster on Cellular Stress Responses in Aging-Associated Diseases, Faculty of Medicine, University of Cologne, Cologne, Germany

^31^ MR-Research in Neurosciences, Department of Cognitive Neurology, University Medical Center Goettingen, Goettingen, Germany

^32^ Berlin Center for Advanced Neuroimaging, Charité University Medicine Berlin, Berlin, Germany

^33^ Department of Neurology, University Hospital Bonn, Bonn, Germany

^34^ Department for Biomedical Magnetic Resonance, University of Tübingen, Tübingen, Germany

^35^ Department of Psychiatry and Psychotherapy, University Hospital Magdeburg, Magdeburg, Germany

^36^ Institute of Diagnostic and Interventional Radiology, Pediatric Radiology and Neuroradiology, University Medical Centre Rostock, Rostock, Germany

^37^ Charité – Universitätsmedizin Berlin, corporate member of Freie Universität Berlin and Humboldt Universität zu Berlin, Experimental and Clinical Research Center (ECRC), Lindenberger Weg 80, 13125 Berlin, Germany.

^38^ German Center for Mental Health (DZPG), Munich, Germany

^39^ Department of Psychiatry and Psychotherapy, Charité – University Medicine Berlin, Berlin, Germany

***Correspondence:**

Martin Dyrba

[martin.dyrba@dzne.de](mailto:martin.dyrba@dzne.de), +493814949482.
German Center for Neurodegenerative Diseases (DZNE), Site Rostock/Greifswald, c/o Zentrum für Nervenheilkunde, Gehlsheimer Str. 20, D-18147 Rostock, Germany.

Devesh Singh

[devesh.singh@med.uni-rostock.de](mailto:devesh.singh@med.uni-rostock.de)
Institute of Diagnostic and Interventional Radiology, Pediatric Radiology and Neuroradiology, University Medical Centre Rostock, Rostock, Germany.

Ernst-Heydemann-Str. 6, 18057 Rostock.

S1 Neuroimaging datasets

| **ADNI** | **CN** | **MCI** | **AD** | **FTD** |
| --- | --- | --- | --- | --- |
| **Age** | 72.48 ± 6.99 | 73.20 ± 7.92 | 74.94 ± 7.90 |  |
| **MMSE** | 29.11 ± 1.13 | 27.70 ± 1.93 | 22.68 ± 3.25 |  |
| **Sex (M/F)** | 239/341 | 224/174 | 142/107 |  |
| **AIBL** |  |  |  |  |
| **Age** | 72.44 ± 6.18 | 74.33 ± 6.85 | 73.18 ± 7.26 |  |
| **MMSE** | 28.73 ± 1.23 | 27.04 ± 2.19 | 21.24 ± 5.31 |  |
| **Sex (M/F)** | 188/260 | 49/47 | 26/36 |  |
| **DELCODE** |  |  |  |  |
| **Age** | 68.25 ± 5.43 | 72.46 ± 5.72 | 74.66 ± 6.24 |  |
| **MMSE** | 29.47 ± 0.83 | 27.80 ± 1.97 | 23.09 ± 3.25 |  |
| **Sex (M/F)** | 120/162 | 81/72 | 42/61 |  |
| **EDSD** |  |  |  |  |
| **Age** | 68.59 ± 6.00 | 71.11 ± 7.41 | 72.69 ± 8.29 |  |
| **MMSE** | 28.61 ± 3.18 | 26.34 ± 2.99 | 20.86 ± 5.28 |  |
| **Sex (M/F)** | 98/97 | 94/76 | 56/78 |  |
| **NIFD** |  |  |  |  |
| **Age** | 62.67 ± 7.25 |  |  | 63.01 ± 7.08 |
| **MMSE** | 29.38 ± 0.77 |  |  | 23.79 ± 7.21 |
| **Sex (M/F)** | 58/74 |  |  | 75/57 |
| **DESCRIBE** |  |  |  |  |
| **Age** | 60.57 ± 13.40 |  |  | 64.08 ± 9.50 |
| **MMSE** | 29.07 ± 1.15 |  |  | 23.04 ± 6.87 |
| **Sex (M/F)** | 29/29 |  |  | 37/24 |
|  |  |  |  |  |
| **NIFD** | **CN** | **BV** | **SV** | **PFNA** |
| **Age** | 62.67 ± 7.25 | 60.97 ± 6.59 | 62.65 ± 6.10 | 67.44 ± 7.29 |
| **MMSE** | 29.38 ± 0.77 | 23.87 ± 5.66 | 22.59 ± 9.57 | 25.03 ± 6.78 |
| **Sex (M/F)** | 58/74 | 42/21 | 21/16 | 12/20 |
| **DESCRIBE** | **CN** | **BV** |  |  |
| **Age** | 60.57 ± 13.40 | 64.08 ± 9.50 |  |  |
| **MMSE** | 29.07 ± 1.15 | 23.04 ± 6.87 |  |  |
| **Sex (M/F)** | 29/29 | 37/24 |  |  |

Supplementary Table S1: Patient statistics separated by the diagnosis group. The statistics are reported for each of the seven data cohorts. The patients were pooled from the following study cohorts: ADNI phase 2 and phase 3, AIBL, DELCODE, DESCRIBE, EDSD, and NIFD. CN: cognitively normal, MCI: mild cognitive impairment, AD: dementia due to Alzheimer’s disease, FTD: Frontotemporal dementia, where phenotypes include, BV: behavioral variant of FTD, SV: semantic variant of FTD, and PFNA: progressive nonfluent aphasia. MMSE: mini-mental state examination score, F: female, M: male. Numbers are reported as (mean ± sd).

S2 Model Training

We trained a DenseNet model, using a stratified five-fold cross-validation (see Supplementary Figure S1). The models were trained for a three-way classification - AD-vs-CN-vs-FTD. Here Alzheimer’s dementia (AD) patients and patients with amnestic mild cognitive impairment (MCI) were merged into one disease-positive class, while multiple phenotypes of frontotemporal dementia (FTD) - behavioral variant (bvFTD), semantic dementia (SD), and progressive nonfluent aphasia (PNFA) were also clubbed under one FTD class. These two classes were compared against the cognitively normal (CN) participants, i.e., the control class.

Categorical cross-entropy was chosen as the loss function. The models were optimized using the Adam optimizer with a learning rate of 0.0001, and other parameter settings were set to default. We trained the models for 100 epochs, using a batch size of 128. To reduce model over-fitting, an early stopping regularization method was applied, monitoring the validation set loss as a performance metric over epochs, with patience of 5 epochs and a minimum change threshold of 0.01. To avoid overfitting, we also weighted the model’s error with the label’s class weight. During each cross-validation run, only the best-performing model was saved.

For training, the data augmentations were generated using AUCMEDI Python package, where 3D volumes were randomly left/right-flipped with a 50% probability, and rescaled with a 50% probability within the zooming in or out limits of 90% and 110%. These augmentations were only applied during model training and were disabled on validation and test sets.


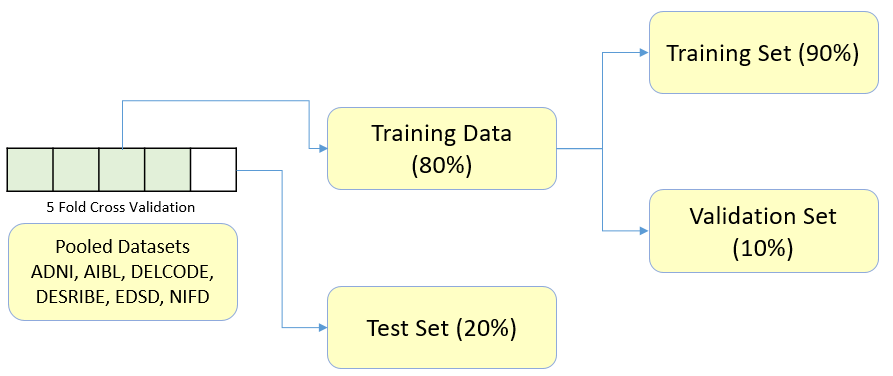


Supplementary Figure S1 Schematics representation of the data splitting for CNN model training.

Based on the results from the 5-fold cross-validation training of the models (see Supplementary Table S2), we chose fold 1 as the default model for further analysis in our study. The model training results from fold 1 are illustrated in Supplementary Figure S2.

| **Fold** | **Acc ADvsCN** | **Acc FTDvsCN** | **Acc ADvsFTD** | **AUC ADvsCN** | **AUC FTDvsCN** | **AUC ADvsFTD** |
| --- | --- | --- | --- | --- | --- | --- |
| 1 | 78.18 | 96.89 | 88.24 | 0.90 | 0.96 | 0.97 |
| 2 | 78.24 | 92.94 | 43.79 | 0.80 | 0.90 | 0.78 |
| 3 | 84.16 | 96.61 | 61.27 | 0.89 | 0.99 | 0.91 |
| 4 | 83.77 | 95.76 | 60.26 | 0.88 | 0.94 | 0.87 |
| 5 | 69.13 | 92.63 | 15.12 | 0.78 | 0.95 | 0.84 |
| Mean(sd) | 78.7±6.07 | 94.97±2.04 | 53.73±26.83 | 0.85±0.05 | 0.95±0.03 | 0.87±0.07 |

Supplementary Table S2: Performance metrics on the test set. Acc: simple accuracy, AUC: Area under the (ROC) curve. CN: cognitively normal, AD: dementia due to Alzheimer’s disease (which due to design choices, also includes the amnestic mild cognitive impairment (MCI) subjects), and FTD: frontotemporal dementia.


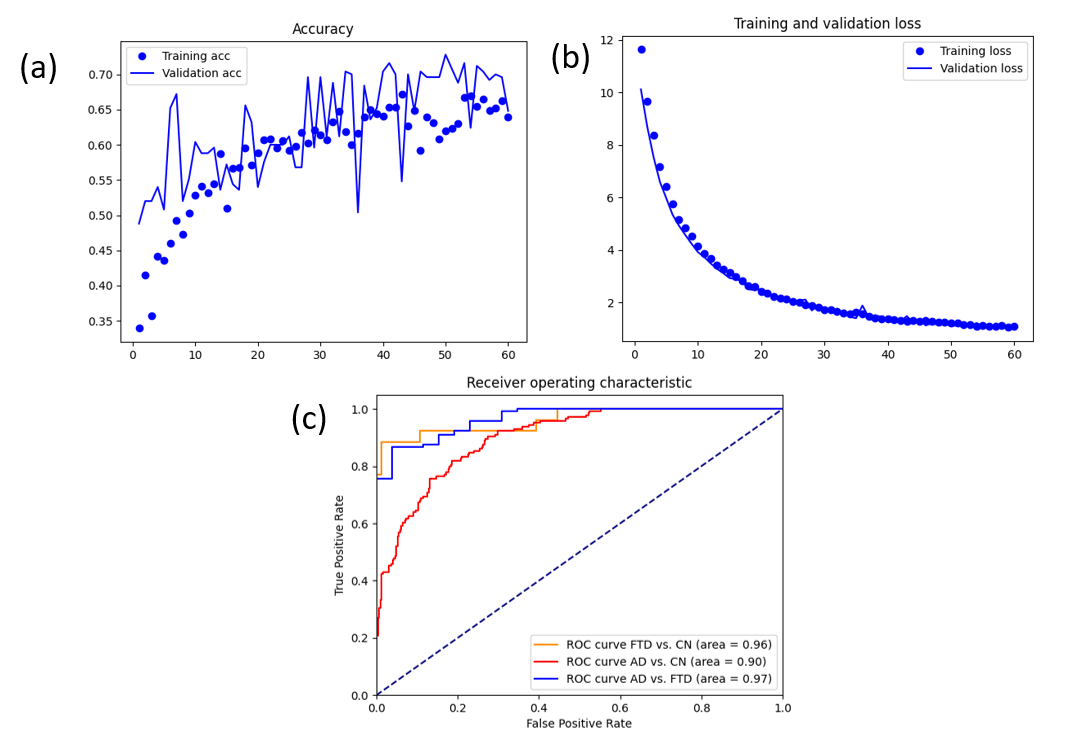


Supplementary Figure S2. Model training results from fold 1: (a) (simple) Accuracy metric on train and validation set, (b) loss metric on train and validation set, and (c) binarized ROC-AUC curves on the test set. CN: cognitively normal, AD: dementia due to Alzheimer’s disease (which due to design choices, also includes the amnestic mild cognitive impairment (MCI) subjects), and FTD: frontotemporal dementia.

The mean relevance maps for the test set of fold 1 are visualized below. For relevance attribution, we employed the compositional LRP rule (𝛼 = 1, 𝛽 = 0), as established in our previous work for generating clinically meaningful explanations^1^. To enhance the signal-to-noise ratio during visualization, we re-scaled the relevance intensities based on the 99.99th percentile (𝑞 = 0.9999) and clipped the resulting values to the range [−1, 1]. A Gaussian smoothing filter with a standard deviation of 0.8 was then applied to further improve interpretability.

The mean relevance maps of Alzheimer's disease (AD) dementia and mild cognitive impairment (MCI) patients appeared visually similar; however, distinct patterns emerged when comparing across disease groups. In the AD group, supplementary figure S3, relevance was concentrated in the hippocampus (slices [-20, -10]) and bilaterally in the thalamus (slices [-30, -20]). In contrast, the frontotemporal dementia (FTD) group, supplementary figure S4, exhibited prominent relevance in the frontal lobes, particularly the right insula and frontal opercular cortex in slice -8, as well as the pregenual anterior cingulate cortex (pACC) in slices [37, 44]. Notably, insular involvement was also reported in our prior study^2^, suggesting consistency across different model training strategies and relevance attribution techniques in identifying clinically relevant brain regions.


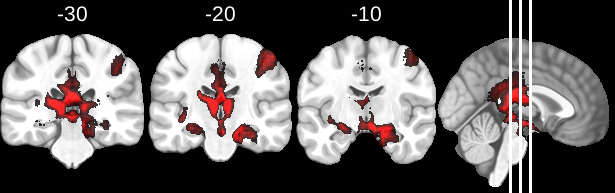


Supplementary Figure S3: Mean relevance maps for the AD group of the test dataset obtained using the LRP𝛼=1, 𝛽=0 relevance propagation method overlaid on MNI brain template. Coronal slices show Y=[-10,-20,-30] mm in MNI reference space are shown. The most relevant input regions are highlighted. Relevance maps were created following proportional scaling of the activations.


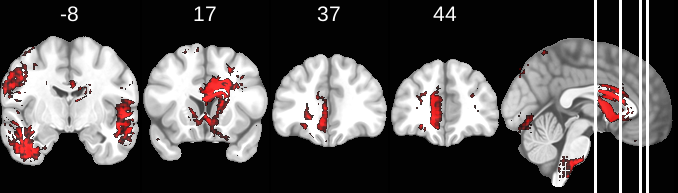


Supplementary Figure S4: Mean relevance maps for the FTD group of the test dataset obtained using the LRP𝛼=1, 𝛽=0 relevance propagation method overlaid on MNI brain template. Coronal slices show Y=[-8,17,37,44] mm in MNI reference space are shown. The most relevant input regions are highlighted. Relevance maps were created following proportional scaling of the activations.

**References**

[1] Dyrba, Martin, Arjun H. Pallath, and Eman N. Marzban. "Comparison of CNN visualization methods to aid model interpretability for detecting Alzheimer’s disease." Bildverarbeitung für die Medizin 2020: Wiesbaden: Springer Fachmedien Wiesbaden, 2020.

[2] Gryshchuk, Vadym, et al. "Contrastive self-supervised learning for neurodegenerative disorder classification." Frontiers in Neuroinformatics 19 (2025): 1527582.

S3 Feature Selection with Mutual Information

All features used in the mutual information analysis were derived from w-scores, representing age, sex, brain size and MRI scanner strength adjusted residualized values. Average cortical thickness measures were only estimated for cortical regions (e.g., superior temporal gyrus, frontal lobe areas) and not for subcortical structures (e.g., hippocampus, thalamus, or basal ganglia), which explains the absence of cortical thickness values in these regions.

To enhance transparency and accessibility, we have uploaded the intermediate results from our analysis pipeline to [GitHub](https://github.com/martindyrba/xai4dementia-framework/tree/master/results). The repository includes a CSV file that specifies, for each of the 120 regions, which of the three feature types (CNN relevance, volumetry, or cortical thickness) passed the mutual information threshold and were included in downstream analysis. This is process outcome is also visualized in Supplementary Figure S5.

**
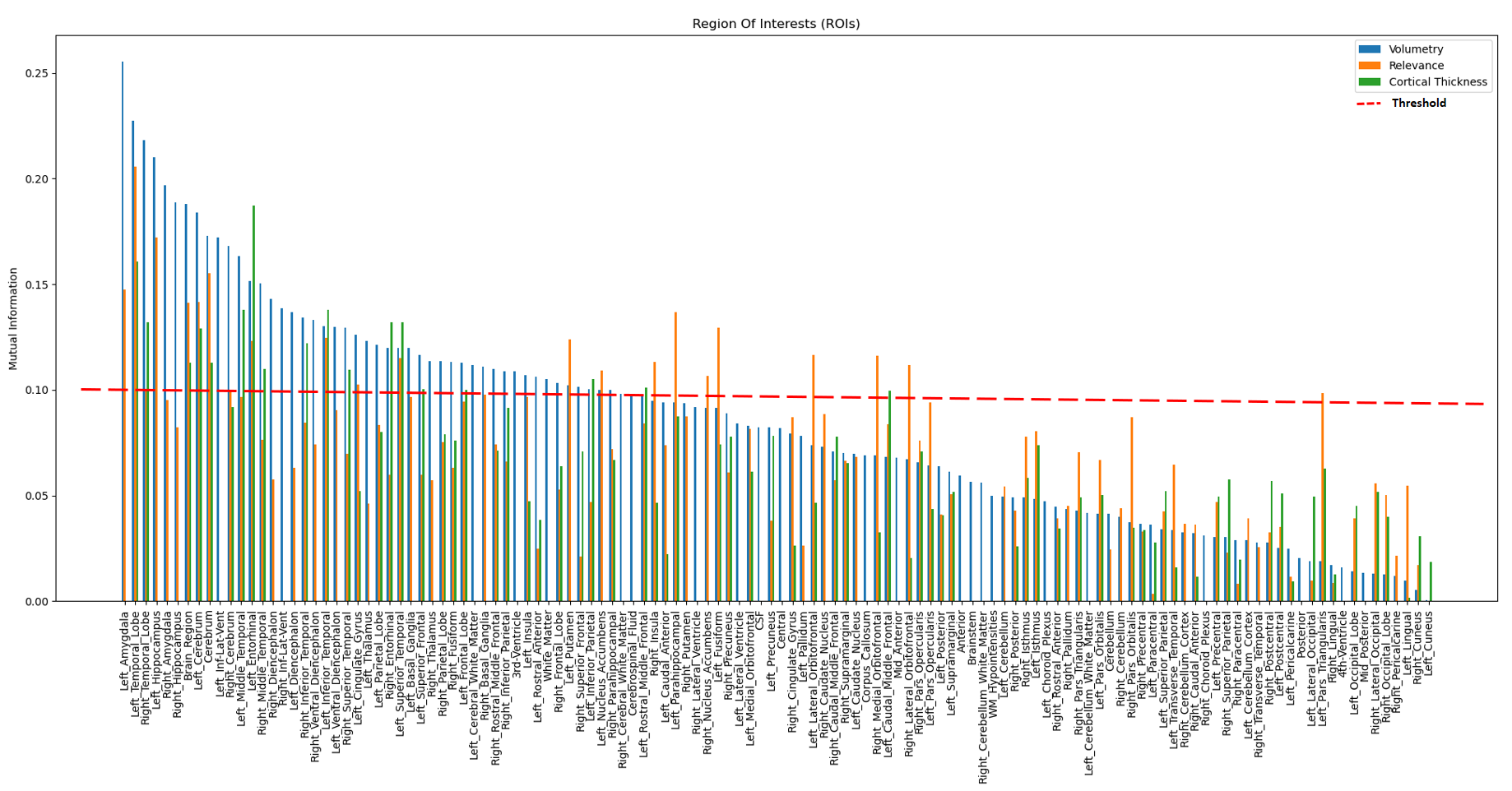
**Supplementary Figure S5: Mutual Information-Based Feature Selection: This figure illustrates the mutual information values computed across three different *w-score* features - CNN relevance, volumetry, and cortical thickness w-scores, representing their shared information content in comparison to the disease diagnosis label. The w-score features were sorted according to their mutual information on the volumetry features. The w-score features with mutual information above the threshold of 0.1 were retained as relevant and were selected for further analysis. For a vector graphic rendering, please refer to the GitHub version of the plot.

S4 Mixed-Effects Models of Cognitive Trajectories

Mixed-effects model experiments were done to investigate cognitive decline in patients, while accounting for repeated measures and inter-individual variability. The analysis was conducted using data from two groups: the Alzheimer’s Disease Neuroimaging Initiative (ADNI) and the DZNE Longitudinal Study on Cognitive Impairment and Dementia (DELCODE) cohorts. These datasets contain repeated cognitive assessments for each patient for up to 6 years, allowing for a longitudinal investigation of cognitive decline.

We tested a series of increasingly complex mixed-effects models. By incrementally adding predictors and interaction terms, we assessed model fit and explanatory power. The best-fitting model was determined using likelihood ratio tests via ANOVA.

**Model 1 (Base Model):** Includes age, sex, and the interaction between cluster membership and follow-up months (FUMonths) while accounting for repeated measures per participant.

CDR_i_ (or MMSE_i_) = β_0_ + β_1_ age_i_ + β_2_ sex_i_ + β_3_ cluster_i_ + β_4_ FUMonths_i_ + β_5_ (cluster × FUMonths)_i_ + u_i_ + ε_i_

where $u_{i} \sim N(0, \sigma_{u}^{2})$ represents the random intercept for each participant, and $\epsilon_{i} \sim N(0, \sigma^{2})$ is the residual error term.

**Model 2 (Expanded Diagnosis Model):** Adds baseline diagnosis as a fixed effect.

CDR_i_ (or MMSE_i_) = β_0_ + β_1_ age_i_ + β_2_ sex_i_ + β_3_ **baseline_diag_i_** + β_4_ cluster_i_ + β_5_ FUMonths_i_ + β_6_ (cluster × FUMonths)_i_ + u_i_ + ε_i_

**Model 3 (Final Model):** Introduces an interaction between baseline diagnosis and follow-up months.

CDR_i_ (or MMSE_i_) = β_0_ + β_1_ age_i_ + β_2_ sex_i_ + β_3_ baseline_diag_i_ + β_4_ cluster_i_ + β_5_ FUMonths_i_ + β_6_ **(baseline_diag × FUMonths)_i_** + β_7_ (cluster × FUMonths) _i_ + u_i_ + ε_i_

To compare model fit, ANOVA tests were performed, evaluating the nested model comparisons. The results indicated that Model 3 provided the best fit, suggesting that the interaction between baseline diagnosis and follow-up time significantly improves the model’s explanatory power. Specifically, for the CDR global (Supplementary Figure S6), the high-risk converter group showed an annual increase of 0.074 points, while the low-risk group remained relatively stable with an increase of 0.007 points per year.


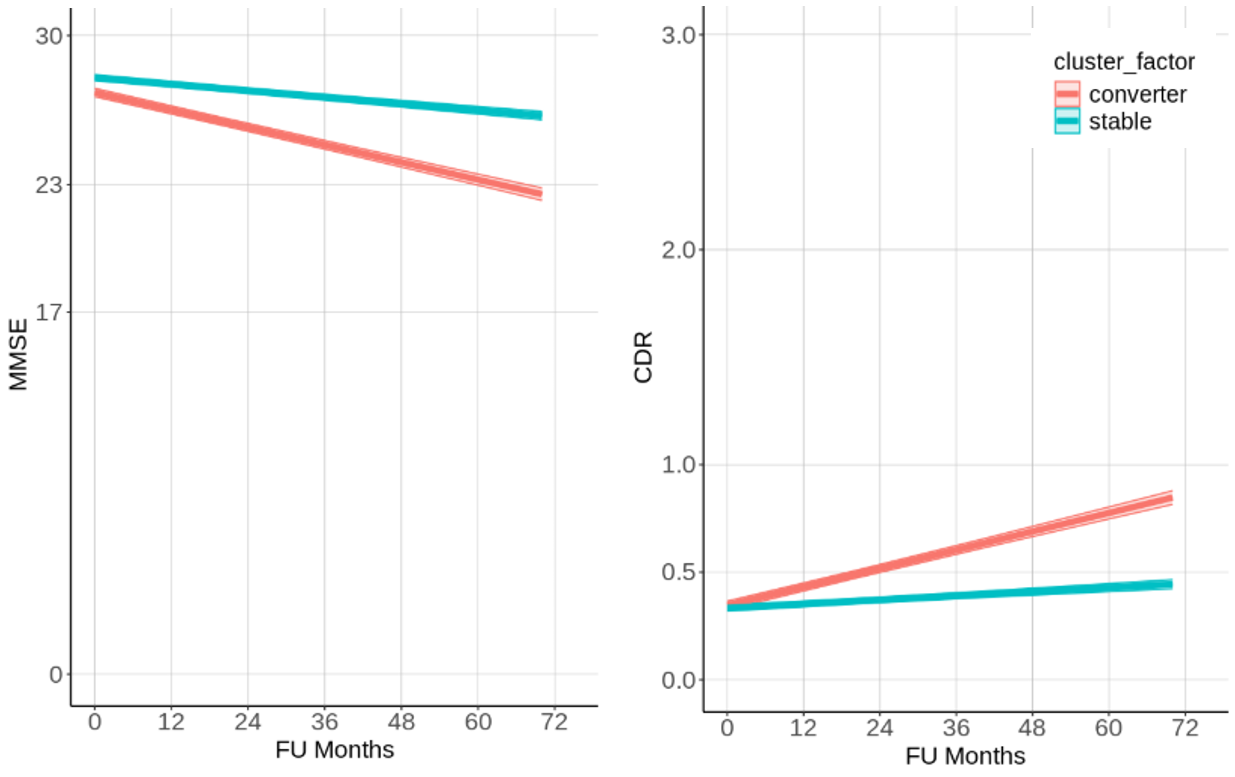


Supplementary Figure S6: Longitudinal cognitive trajectories of different clusters of patients. Values on Clinical Dementia Rating (CDR) global are obtained from mixed effects regression models which included the age, sex, baseline disease diagnosis, and the interaction between cluster membership and follow-up time in months (FU Months), as well as the interaction between baseline disease diagnosis and follow-up months. The model also included random intercepts for each patient to account for repeated measurements. The shaded regions represent 95% confidence intervals.

S5 Explanation-by-example plots for Clinical Dementia Rating (CDR)


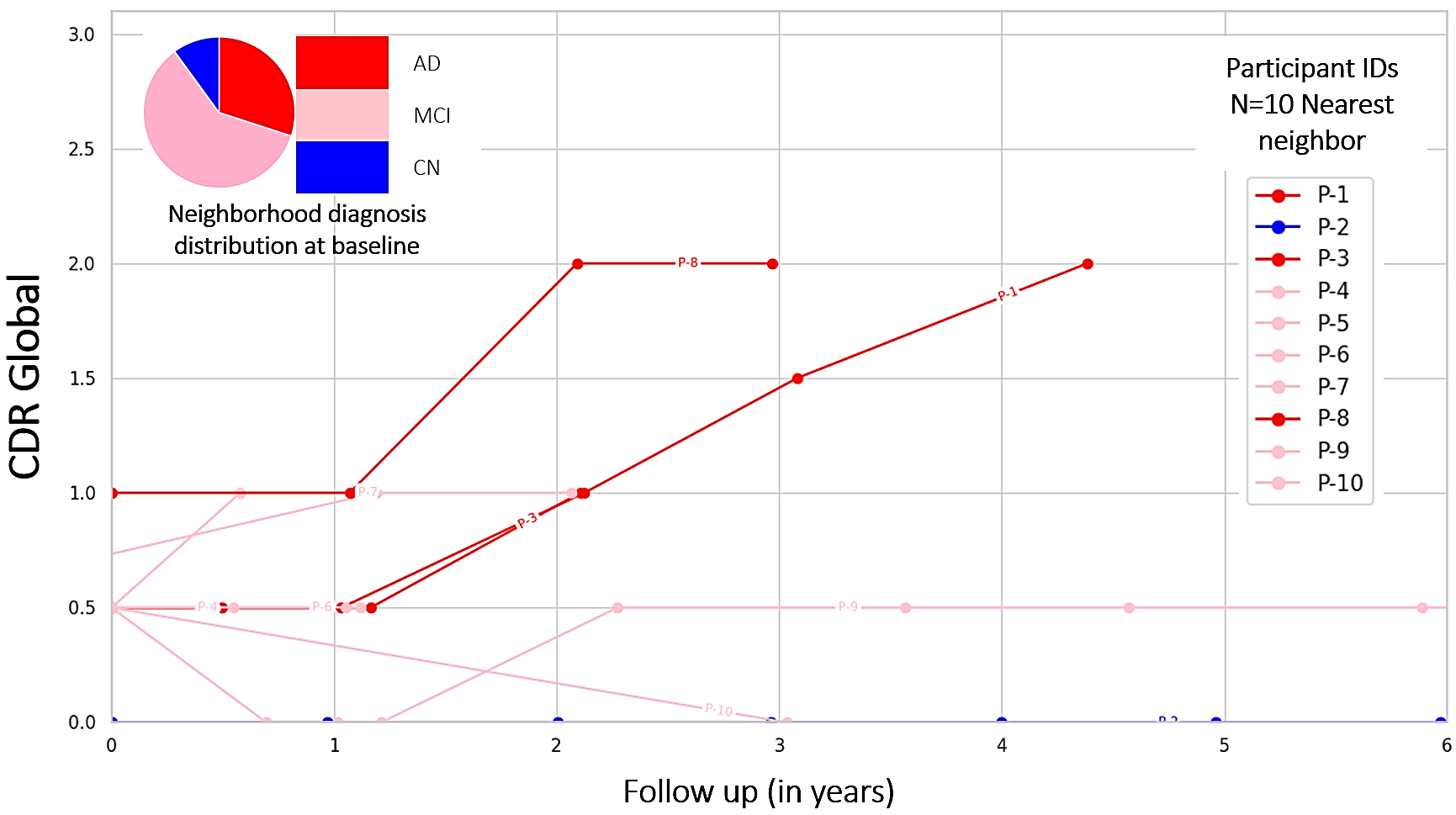


Supplementary Figure S7: Explanation-by-examples: Within the context-enriched explanation space, the longitudinal cognitive trajectories of k=10 nearest neighbors of a query patient, from the DELCODE cohort, are shown. Patient IDs of the nearest neighbors are pseudonymised, and the nearest neighbors are listed in the order of increasing Euclidian distance from the query sample. Scores on the cognitive test Clinical Dementia Rating (CDR) global were observed on follow-up examinations for up to 6 years. The cognition trajectories are additionally color-coded by the baseline disease diagnosis.

S6 Explanation-by-example plots, each participant colored individually


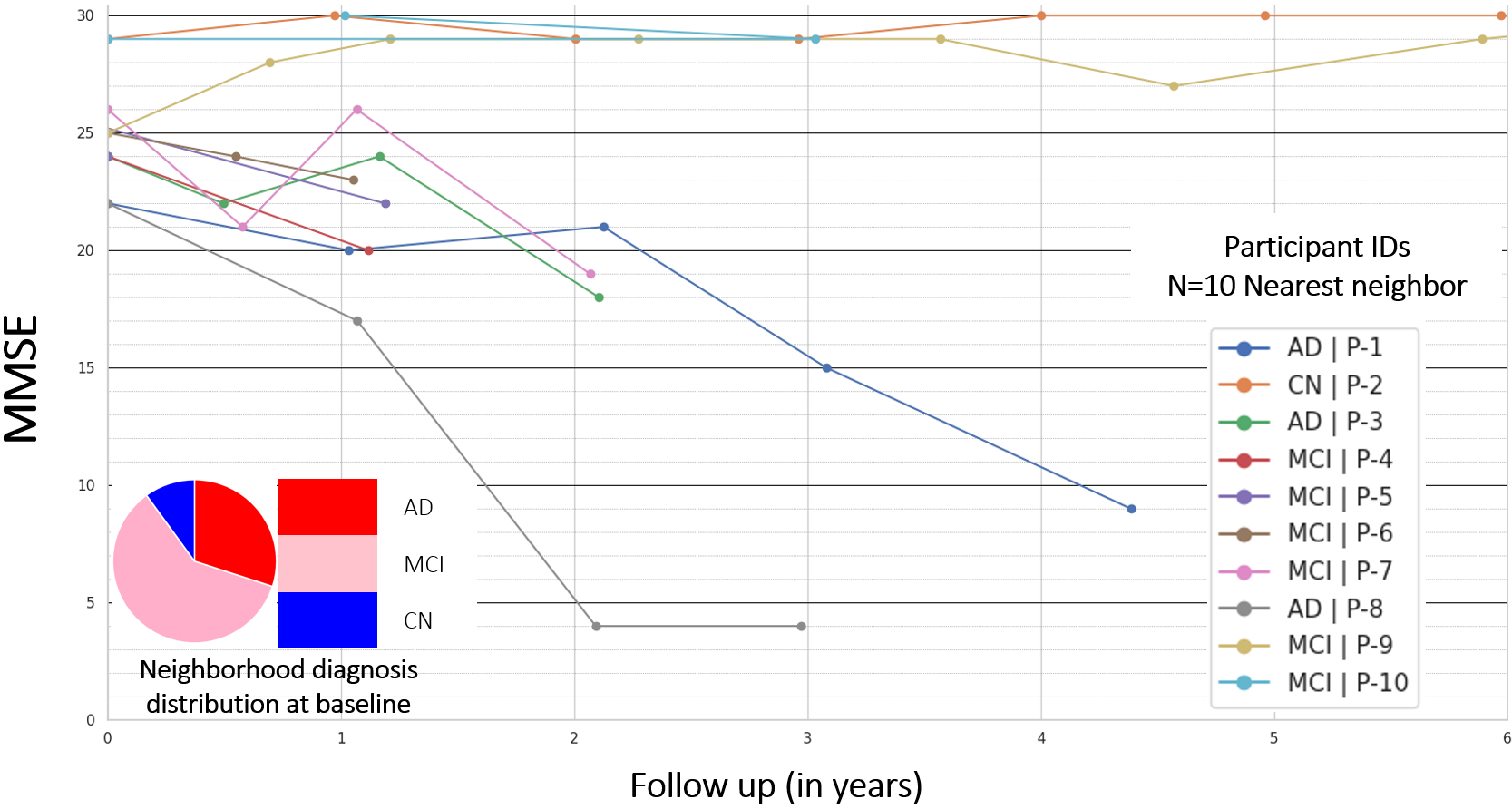
 Supplementary Figure S8**:** Explanation-by-examples: Longitudinal MMSE trajectories of the k = 10 nearest neighbors of a query participant from the DELCODE cohort, observed for up to 6 years. Here, each cognitive trajectory is shown in a unique color for more detail.


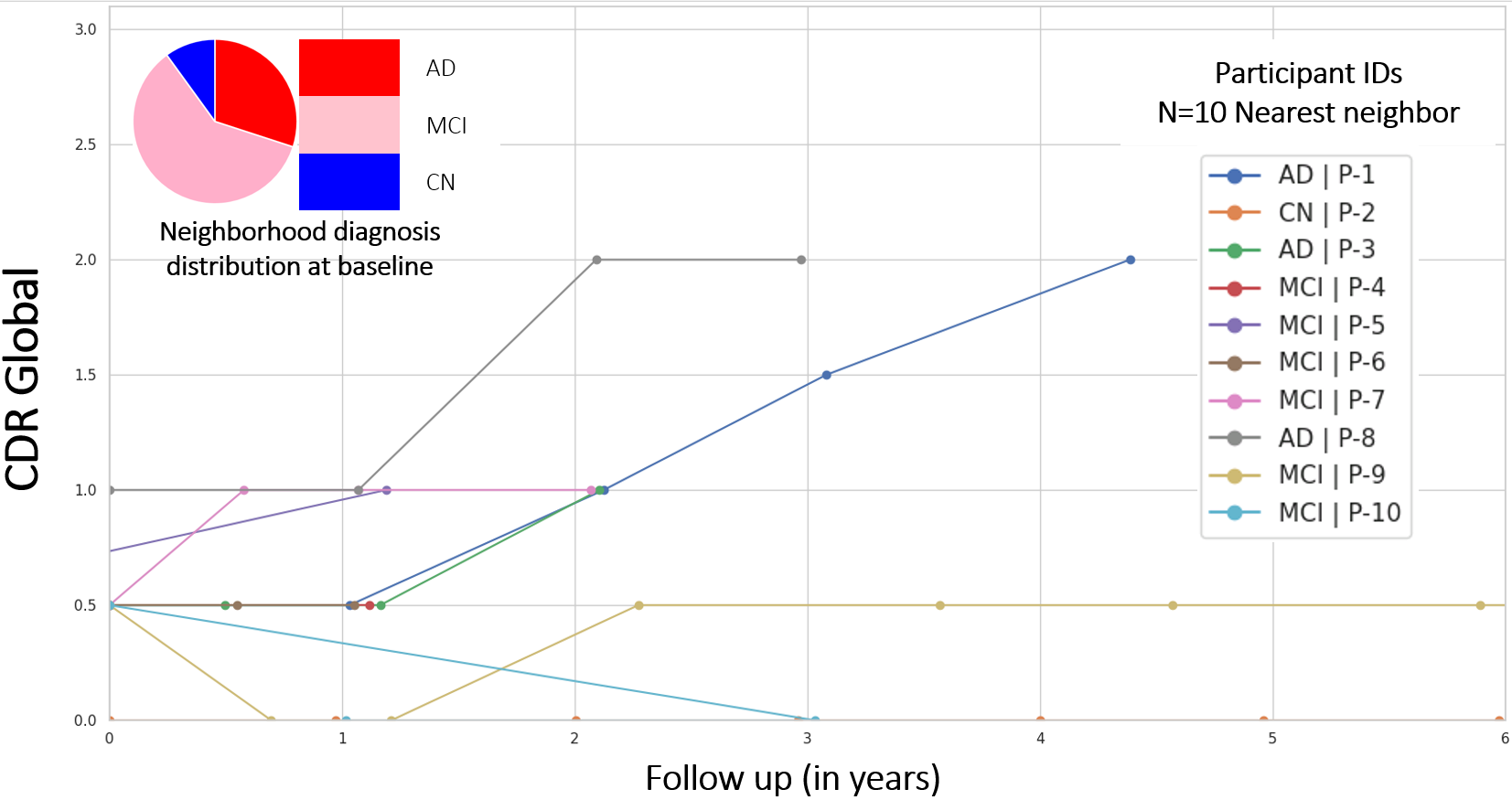


Supplementary Figure S9**:** Explanation-by-examples: Longitudinal CDR trajectories of the k = 10 nearest neighbors of a query participant from the DELCODE cohort, observed for up to 6 years. Here, each cognitive trajectory is shown in a unique color for more detail.
